# CXCL10 levels at hospital admission predict COVID-19 outcome: hierarchical assessment of 53 putative inflammatory biomarkers in an observational study

**DOI:** 10.1186/s10020-021-00390-4

**Published:** 2021-10-18

**Authors:** Nicola I. Lorè, Rebecca De Lorenzo, Paola M. V. Rancoita, Federica Cugnata, Alessandra Agresti, Francesco Benedetti, Marco E. Bianchi, Chiara Bonini, Annalisa Capobianco, Caterina Conte, Angelo Corti, Roberto Furlan, Paola Mantegani, Norma Maugeri, Clara Sciorati, Fabio Saliu, Laura Silvestri, Cristina Tresoldi, Bio Angels for COVID-BioB Study Group, Nicola Farina, Luigi De Filippo, Marco Battista, Domenico Grosso, Francesca Gorgoni, Carlo Di Biase, Alessio Grazioli Moretti, Lucio Granata, Filippo Bonaldi, Giulia Bettinelli, Elena Delmastro, Damiano Salvato, Giulia Magni, Monica Avino, Paolo Betti, Romina Bucci, Iulia Dumoa, Simona Bossolasco, Federica Morselli, Fabio Ciceri, Patrizia Rovere-Querini, Clelia Di Serio, Daniela M. Cirillo, Angelo A. Manfredi

**Affiliations:** 1 Division of Immunology, Transplantation and Infectious Diseases, IRCCS San Raffaele Scientific Institute, Via Olgettina 60, 20132 Milano, Italy; 2 Vita-Salute San Raffaele University, Milano, Italy; 3 Emerging Bacterial Pathogens Unit, IRCCS San Raffaele Scientific Institute, Milano, Italy; 4 University Centre for Statistics in the Biomedical Sciences (CUSSB), Vita-Salute San Raffaele University, Milan, Italy; 5 Division of Genetics and Cell Biology, IRCCS San Raffaele Scientific Institute, Milano, Italy; 6 Division of Neuroscience, IRCCS San Raffaele Scientific Institute, Milano, Italy; 7 Division of Experimental Oncology, IRCCS San Raffaele Scientific Institute, Milano, Italy; 8 Hematology and Bone Marrow Transplant, IRCCS San Raffaele Scientific Institute, Milano, Italy; 9 Faculty of Biomedical Sciences, Swiss University, Lugano, Switzerland

**Keywords:** COVID-19 severity predictors, Biomarkers, Decision tree, CXCL10

## Abstract

**Background:**

Host inflammation contributes to determine whether SARS-CoV-2 infection causes mild or life-threatening disease. Tools are needed for early risk assessment.

**Methods:**

We studied in 111 COVID-19 patients prospectively followed at a single reference Hospital fifty-three potential biomarkers including alarmins, cytokines, adipocytokines and growth factors, humoral innate immune and neuroendocrine molecules and regulators of iron metabolism. Biomarkers at hospital admission together with age, degree of hypoxia, neutrophil to lymphocyte ratio (NLR), lactate dehydrogenase (LDH), C-reactive protein (CRP) and creatinine were analysed within a data-driven approach to classify patients with respect to survival and ICU outcomes. Classification and regression tree (CART) models were used to identify prognostic biomarkers.

**Results:**

Among the fifty-three potential biomarkers, the classification tree analysis selected CXCL10 at hospital admission, in combination with NLR and time from onset, as the best predictor of ICU transfer (AUC [95% CI] = 0.8374 [0.6233–0.8435]), while it was selected alone to predict death (AUC [95% CI] = 0.7334 [0.7547–0.9201]). CXCL10 concentration abated in COVID-19 survivors after healing and discharge from the hospital.

**Conclusions:**

CXCL10 results from a data-driven analysis, that accounts for presence of confounding factors, as the most robust predictive biomarker of patient outcome in COVID-19.

**Graphic abstract:**

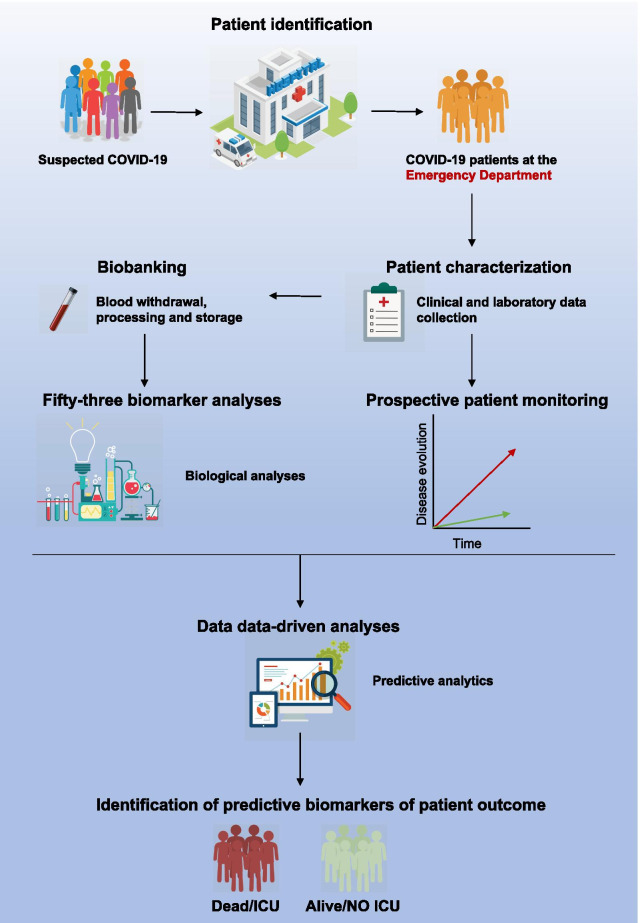

**Supplementary Information:**

The online version contains supplementary material available at 10.1186/s10020-021-00390-4.

## Introduction

The coronavirus disease 2019 (COVID-19) pandemic caused by severe acute respiratory syndrome coronavirus 2 (SARS-CoV-2) infection threatens healthcare systems around the world and has killed more than two and a half millions of people worldwide. Clinical manifestations are heterogeneous, the spectrum of severity ranging from self-limiting to life-threatening disease. The fragmented data collected during the initial emergency limited the possibility to investigate the effect of highly correlated covariates and to model the interplay between risk factors biomarkers and outcomes by means of standard statistical approaches. However, it is mandatory to find statistical approaches to COVID 19 case series data that allow for an early identification of patients at increased risk of adverse outcome, such as death or transfer to intensive care unit (ICU). Patient stratification would allow for an appropriate allocation of available resources and selection of the intensity of care.

The heterogeneity in clinical outcome is influenced by the individual host response to SARS-CoV-2 infection. Better outcomes might reflect an effective early innate immune response to the primary infection, that limits the collateral damage to peripheral tissues caused by unrestrained inflammation (Li, et al. 2020; Kuri-Cervantes, et al. 2020; Valle et al. 2020; Arunachalam et al. 2020; Laing et al. 2020). A poor outcome in contrast might depend on a failure of the early immune response to clear the virus, leading to cell and tissue damage, self-amplifying the release of endogenous adjuvants and alarmins that further trigger the unrestrained production of inflammatory cytokines and chemokines in non-surviving patients. In either case, SARS-CoV-2 infection elicits an acute phase response. Median times from symptom onset to viral clearance in surviving or deceased patients are 20 and 19 days, respectively (Zhou et al. 2020). This kinetics strongly implies a role for acquired immune responses. Indeed, the coordinated activation of SARS-CoV-2–specific memory T and B cells together with the generation of SARS-CoV-2-specific high-affinity antibodies appears to be protective, while non-synchronized immune responses fail to limit the infection with maladaptive paroxysmal activation of the inflammatory and coagulative cascades (Dan et al. 2021; Rydyznski Moderbacher 2020).

Several candidate markers, routinely used in clinical practice or selected based on their biological action, have been associated with disease severity and in some cases with worse clinical outcomes (Chi et al. 2020; Hue et al. 2020; Chen et al. 2020; Bulow Anderberg 2021; Yang 2020; Danwang et al. 2020; Tian et al. 2020; Lucas et al. 2020). Standard statistical models to support clinical decision-making combine the results of demographic characteristics, clinical information, imaging techniques and selected biomarkers. Not surprisingly, given the redundancy in the inflammatory factors generated during the acute phase response, it is difficult to impartially identify which signals could provide a specific advantage over those already commonly used in the clinical routine.

Decision trees are classification algorithms used to identify models predicting binary outcomes (Westreich et al. 2010; Pauker and Kassirer 1987; Detsky et al. 1997) and may be used either in an exploratory fashion or in a predictive way (Siciliano 1998; Siciliano et al. 2008). We have used the classification and regression tree (CART) in both ways, in order to identify those variables among patient characteristics and fifty-three innovative molecular biomarkers evaluated at hospital admission (and corresponding cut-offs) that could allow to better discriminate patients with respect to their survival outcome and ICU entry.

## Methods

### Patients and study design

This retrospective and prospective investigation is included in the more extensive COVID-BioB study, a large observational study conducted at the San Raffaele University Hospital, a tertiary health-care center in Milan, Italy. All adult patients (age ≥ 18 years) admitted to San Raffaele University Hospital for COVID-19 from 25 February 2020 were enrolled in the COVID-BioB study (Ciceri 2020). COVID-19 diagnosis was confirmed by a positive SARS-CoV-2 real-time reverse-transcriptase polymerase chain reaction (RT-PCR) from a nasopharyngeal swab in the presence of clinical and/or radiologic findings suggestive of COVID-19 pneumonia. Clinical findings suggestive of COVID-19 were new-onset fever and/or respiratory tract symptoms (eg, cough, dyspnea). Radiologic findings were investigated through chest X-rays and/or chest CT scan. Signs of interstitial pneumonia, ground glass opacities or crazy-paving pattern with or without parenchymal consolidations were considered suggestive of COVID-19 pneumonia according to previous reports (Guneyli et al. 2020). As part of the COVID-BioB protocol, blood samples from all enrolled patients were collected and stored in the COVID-19 biobank of our Institution according to appropriate quality control systems (Rovere-Querini 2020). Biologic specimens were complemented by detailed demographic, laboratory and clinical data recorded in a dedicated electronic case record form (eCRF). One hundred eleven patients evaluated at our Institution at our Institution during the first wave of the pandemic between March 18th and May 5th, 2020 were included in the present analysis. Median (IQR) time that elapsed between hospital admission and venepuncture was 1 (0–2) days. For survivors, samples were obtained both at admission and after viral clearance and hospital discharge during routinely scheduled evaluations in a dedicated outpatient Clinics (Farina 2020; Lorenzo 2020; Rovere Querini et al. 2020). The COVID-BioB study protocol, compliant with the declaration of Helsinki, was approved by the Hospital Ethics Committee (protocol no. 34/int/2020) and registered on ClinicalTrials.gov (NCT04318366). All patients signed informed consent.

### Biomarkers

Plasma-EDTA was obtained by centrifugation of venous blood, immediately frozen and maintained at -80 °C until subsequent analyses. Plasma was inactivated using tri-(n-butyl) phosphate and Triton X-100 (Sigma) (0.3% and 1%, respectively) for 2 h (Darnell and Taylor 2006). A panel of 53 putative biomarkers was analyzed (Additional file 1: Table S1). Validated biomarkers routinely used in patients care that are associated to COVID-19 severity and might predict outcome were assessed, including concentration of C-reactive protein, creatinine and LDH (Danwang et al. 2020; Tian et al. 2020; Ji 2020) and assessment of the NLR (Simadibrata et al. 2021). The PaO^2^/FiO^2^ ratio was used as a surrogate marker of hypoxia (< 300 mm Hg). The concentration in the plasma of HMGB1, H3 histones and neutrophil gelatinase-associated lipocalin (NGAL), ficolin-3, C1q, C4d, C3a, C5a, mannose-binding lectin, MBL; MBL-associated serine proteases (MASP-) 1 and 2; protein 1 similar to chitinase-3, YKL-40, T-cell immunoglobulin and mucin domain 1, TIM-1, anti-spike S1 SARS-CoV-2 IgGs, IL-18, adiponectin, leptin, hepcidin were measured by commercial ELISA. was assessed using commercial ELISAs (HMGB1 ELISA, ST51011 TECAN; GDMBS937951 Human Histone Cluster 1 HIST1H3A, Valter Occhiena; NGAL, DY1757 R&D DuoSet ELISA Development System; S1 SARS-CoV-2 IgGs, Euroimmun, PerkinElmer company; Ficolin 3, ab213779 Abcam; Hum C1q BMS2099, Invitrogen; Human Complement Fragment 4d, EKX-UF2R8I-96, Nordic BioSite AB; C3a, BMS2089 Invitrogen; C5a BMS2088 Invitrogen; MBL ELH-MBL-1, Raybiotech; MASP-1 EKX-B932SF-96, Nordic BioSite AB; MASP-2 EKX-JYF1T7-96; Nordic BioSite AB; HumanYLL-40 (CHI9L1) BMS2322,Invitrogen; TIM-1, DY1750B R&D DuoSet ELISA Development System; IL-18, DY318 R&D DuoSet ELISA Development System; adiponectin and leptin ab99968 Abcam and ab179884 Abcam, Intrinsic Hepcidin IDx™ ELISA Kit (Intrinsic Lifesciences); the concentration of neurofilament light chain, NfL; total tau protein; ubiquitin carboxyl-terminal hydrolase L1, UCH-L1; glial fibrillary acidic protein, GFAP was assessed using the Simoa Human Neurology 4-Plex A assay (N4PA, Quanterix). To assess the activation of the neuroendocrine system the concentration of full-length chromogranin A CgA (CgA439), CgA fragments lacking the C-terminal region (CgA-439, 436/39) and the N-terminal fragment, CgA1-76 (vasostatin-1, VS-1) were assessed by sandwich ELISAs, performed and calibrated as described (Tombetti et al. 2016). Multiplex immunoassays (Bio-Rad) based on Luminex technology were used for the quantification of 27 biomarkers among cytokines, chemokines and growth factors in human samples, according to the manufacturer’s instructions (Bio-Plex Pro™ Human Cytokine 27-plex). Data were measured on a Bio-Plex 200 System and calculated using Bio-Plex Manager 6.0 and 6.1 software. Plasma iron concentration was assessed by the “Fe” kit (Randox Laboratories ltd, Crumlin, UK). CRP was assessed by a latex-enhanced immuno-turbidimetric assay (ADVIA Chemistry System, Bayer AG, Leverkusen Germany), serum LDH and creatinine using a COBAS C 8000 modular technology (Roche).

### Statistical analysis

In case of numerical variables, comparisons between independent groups were performed by means of the Mann–Whitney test, while in case of categorical variables with Fisher’s exact test. Comparisons of numerical variables between paired data were performed with paired Wilcoxon test. Spearman’s correlation coefficient was employed to assess the pairwise correlation between numerical variables. To identify clusters of co-expressed biomarkers, hierarchical cluster analysis was applied on the corresponding correlation matrix. In all analyses with multiple testing, false discovery rate (FDR) correction was applied.

For identifying early biomarkers at hospital presentation predicting adverse outcomes (transfer to ICU and death), classification tree models were estimated through the classification and regression tree (CART) method. CART models allow to classify subjects into various risk categories. The method is based on recursive partitioning, a non-parametric statistical approach that uses a series of dichotomous splits, *e.g.* presence or absence of symptoms and other clinical and demographic variables, to create a decision tree, with the goal of correctly classifying members subjects.

Due to the high number of biomarkers considered (fifty-three), before estimating the tree model, a variable selection procedure based on Random Forest was applied to obtain a first reduction of the number of variables. This strategy allows to achieve more robust results with the CART even in presence of a high number of variables and possible intervariable correlations. While the CART method handles the presence of missing data, the Random Forest algorithm cannot and needs the imputation of missing data for using the entire dataset. Therefore, variable selection procedure consisted in: (1) imputing 50 times the missing data; (2) estimating a Random Forest and applying the Boruta algorithm for variable selection (Kursa 2010) on each complete dataset; (3) considering for successive analysis only biomarkers selected in at least 50% of the times.

After the variable selection step, all selected biomarkers together with patient’s characteristics were considered for the estimation of the classification tree. Specifically, we included in both models: sex, comorbidities (hypertension, coronary artery disease and diabetes mellitus), time from symptom onset (TfSO) and NLR. In the model for the prediction of death, we included also: age, degree of hypoxemia (PaO_2_/FiO_2_), concentration of CRP, LDH, and creatinine. The CART algorithm was applied to the entire dataset through the use of surrogate splits for the estimation of the model. Goodness of model prediction was measured through the area under the curve (AUC) of the receiving operator characteristic (ROC) curve of the predicted probabilities.

Kaplan–Meier estimator was used to estimate overall survival curves and logrank test was employed for comparing survival curves between the two groups defined in the classification tree estimated for predicting death.

The significant level was set to 0.05 for all analysis. All analyses were performed with R 4.0.2 (https://www.r-project.org/).

## Results

Demographics, comorbidities, disease characteristics at hospital admission and clinical outcomes of the one hundred eleven patients with COVID-19 included in the present analysis are reported in Table 1. Blood was obtained upon written informed consent after a median (IQR) time from hospital admission of 1 (0–2) days. At sampling, 81 (73%) patients had not received any treatment. Seven (6%) patients had started hydroxychloroquine, 2 (2%) steroids, 1 (1%) lopinavir/ritonavir, 4 (4%) low molecular weight heparin and 16 (14%) a combination of the latter agents for a median time of 1 (0–2) days.

**Table 1:** Sample characteristics

	Total	Patients without ICU (n = 75)	Patients with ICU (n = 36)	p-value	Alive (n = 89)	Dead (n = 22)	p-value
Age, median [IQR]—yr	57.6 [48.5, 66.3]	53.8 [46.4, 63.8]	61.8 [55.3, 69.1]	0.0336^†^	55.5 [48.3, 63.3]	64.4 [59.3, 74.9]	0.0059^†^
Male sex – no. (%)	70 (63.1%)	40 (53.3%)	30 (83.3%)	0.0071^§^	56 (62.9%)	14 (63.6%)	1^§^
Hypertension (HTN)—no. (%)	38 (34.2%)	25 (33.3%)	13 (36.1%)	0.8324^§^	28 (31.5%)	10 (45.5%)	0.2664^§^
Coronary artery disease (CAD)—no. (%)	9 (8.1%)	3 (4%)	6 (16.7%)	0.0671^§^	4 (4.5%)	5 (22.7%)	0.0249^§^
Diabetes mellitus (DM) – no. (%)	22 (19.8%)	10 (13.3%)	12 (33.3%)	0.0336^§^	13 (14.6%)	9 (40.9%)	0.0249^§^
Chronic obstructive pulmonary disease (COPD)—no. (%)	0	0	0	/	0	0	/
Chronic kidney disease (CKD)—no. (%)	4 (3.6%)	1	3	/	3	1	/
Neoplasia—no. (%)	2 (1.8%)	2	0	/	2	0	/
PaO_2_/FiO_2_, median [IQR]	288.57, [201.67 367.6]	319.0 [271.4, 393.3]	195.2 [82.5, 271.3]	< 0.0001^†^	309.5 [228.6, 374.3]	201.7 [131.7, 276.8]	0.0059^†^
NLR, median [IQR]	5.33, [3.3, 8]	4.4444 [2.9, 6.8]	7.7083 [5.4, 10.6]	0.0003^†^	4.8231 [3.2, 7.5]	6.5 [5.4, 13.4]	0.0253^†^
LDH, median [IQR]	374, [273.5, 522.25]	321 [241, 455]	503 [369, 675]	0.0001^†^	334 [252, 483.5]	503 [369, 562]	0.0059^†^
CRP, median [IQR]	85, [24.2, 152.8]	60.1 [9.1, 127.4]	132.5 [69.5, 235.7]	0.0001^†^	76 [16.7, 142.7]	127.1 [57.6, 207.2]	0.0249^†^
Creatinine, median [IQR]	0.89, [0.73, 1.12]	0.8 [0.72, 1]	0.985 [0.85, 1.3]	0.0105^†^	0.82 [0.72, 1.01]	1.2 [0.92, 1.42]	0.0059^†^
Time (Days) from symptom onset to blood draw, median [IQR]	8 [5; 11]	8 [4.5; 11]	8 [6; 10.25]	0.8324^†^	9 [6; 11]	7 [2.25; 9]	0.0651^†^
Time (Days) from hospital admission to blood draw, median [IQR]	1 [0; 2]	1 [0; 1]	1 [1; 2]	0.065^†^	1 [0; 2]	1 [0; 2]	1^†^
Transfer to ICU—no. (%)	36 [32.4%]						
Death—no. (%)	22 [19.8%]						
Time (Days) from hospital discharge to the follow-up sampling, median [IQR]	23 [20–40]						
Total number of Leukocytes median [IQR]	7,2 [5.4–10.3] × 10^9 /L						
Total number of platelets median [IQR]	235 [165–329] × 10^9 /L						

^†^Mann–Whitney test with false discovery rate (FDR) correction (Patients without ICU vs Patients with ICU; Alive vs Dead)

^§^Fisher's test with false discovery rate (FDR) correction (Patients without ICU vs Patients with ICU; Alive vs Dead)

*IQR* interquartile range

*NLR* neutrophils to lymphocytes ratio

*LDH* lactate dehydrogenase

*CRP* C-reactive protein

The majority of patients were males (63.1%) and median (IQR) age was 57.63 (48.46, 66.30) years. Hypertension was the most frequent comorbidity, being present in more than one third of patients (n = 38, 34.2%), followed by diabetes mellitus (n = 22, 19.8%) and coronary artery disease (n = 9, 8.1%). Table 1 also reports the ratio of arterial oxygen partial pressure (PaO_2_) in mmHg to fractional inspired oxygen (FiO_2_) expressed as a fraction (PaO_2_/FiO_2_) which reflects the degree of hypoxemia, and markers such as the NLR and levels of CRP, LDH and creatinine, which are commonly used in the clinics to stratify patients based on disease severity and overall risk.

Thirty-six (32.4%) patients required transfer to the Intensive Care Unit (ICU). 22 (19.8%) patients died of COVID-19 or related complications. Median (IQR) time from Hospital admission to transfer to ICU or to death was 5.5 (1–8) and 15.5 (9.75–29) days, respectively. As expected, patients with worse outcomes were older and more hypoxemic (Table 1). Conventional parameters routinely used to define COVID-19 severity, including NLR, CRP and LDH, and creatinine were also different between survivors and non-survivors or patients transferred or not to ICU (Table 1). The groups did not differ significantly in terms of time elapsed from the onset of symptoms (TfSO) (Mann–Whitney test p = 0.8324 for ICU/non-ICU and p = 0.0651 for dead/alive). Median [IQR] time elapsed from the onset of symptoms were: 8 days (4.5; 11) for patients transferred to ICU, 8 days [6; 10.25] for patients not transferred to ICU, 9 day (6; 11) for survivors and 7 days (2.25; 9) for non-survivors patients (Table 1).

**Fig. 1:**
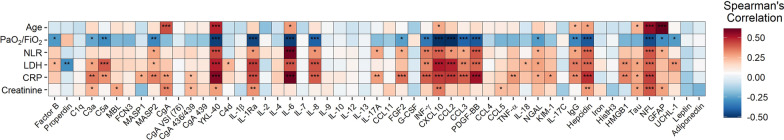
Spearman’s correlations between clinical characteristics and biomarker levels. The magnitude of each correlation is denoted with a color, whereby the red color indicates a positive correlation and blue color represents a negative correlation, such that the deeper the color, the stronger is the correlation. Levels of statistical significance with false discovery rate (FDR) correction are denoted as: p < 0.05, *p < 0.01, ***p < 0.001. IgG = anti-SARS-CoV2 spike 1 IgGs

Many of the fifty-three putative biomarkers, which include signals of cell and tissue injury, markers of innate humoral immune response activation, cytokines, chemokines and adipocytokines, the iron metabolism regulator hepcidin and neuroendocrine molecules, significantly correlated with the degree of hypoxia, NLR, concentration of CRP and LDH, and creatinine (Fig. 1).

**Fig. 2:**
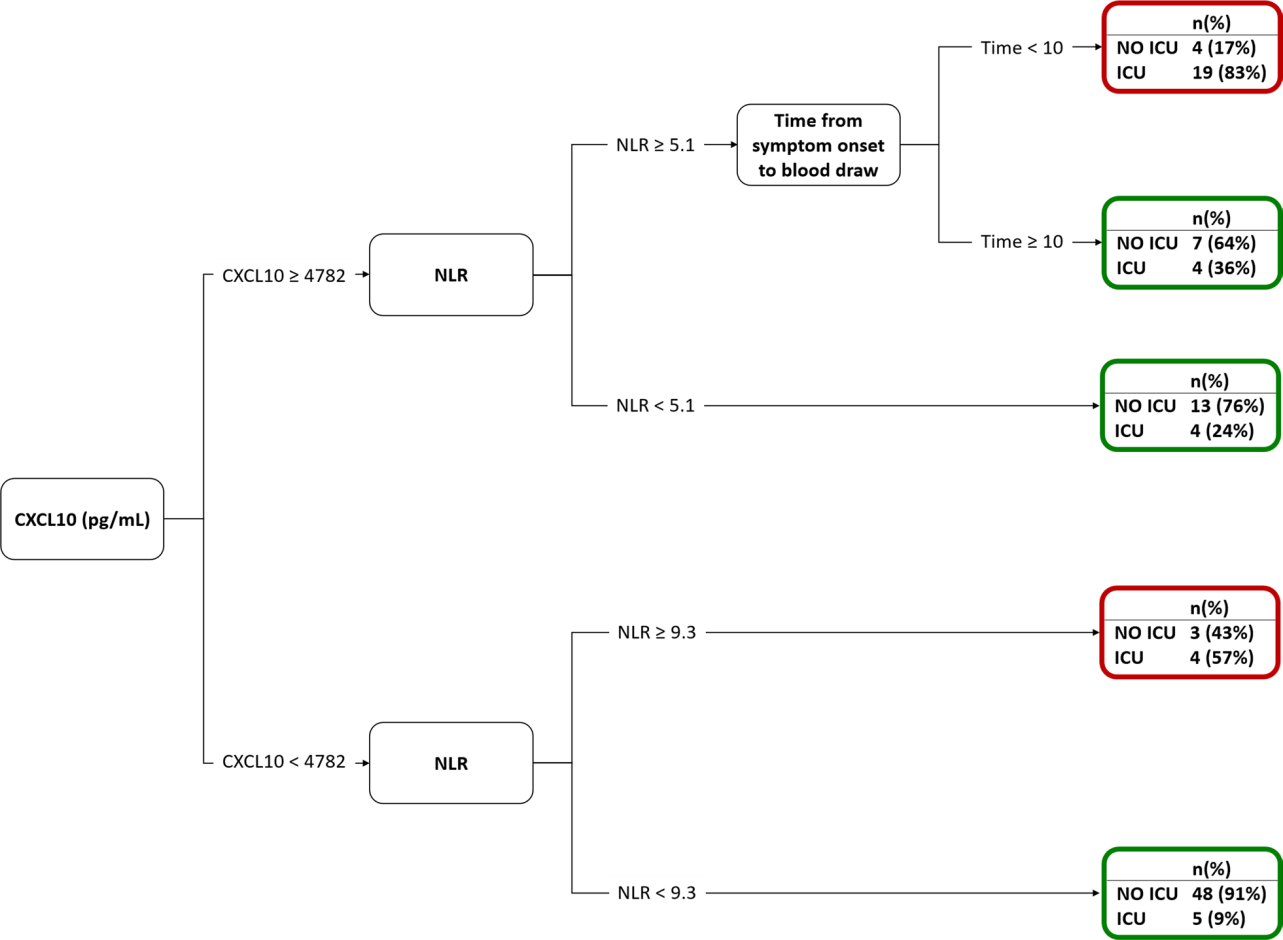
Decision tree for the prediction of the ICU admission (AUC [95% CI] = 0.8374 [0.6233–0.8435]). NLR = neutrophils to lymphocytes ratio

**Fig. 3:**
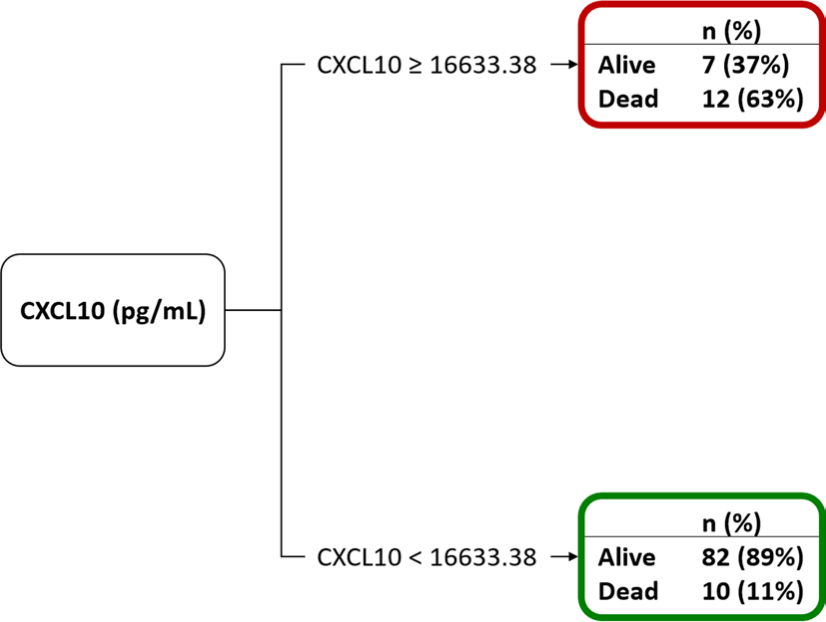
Decision tree for the prediction of death (AUC [95% CI] = 0.7334 [0.7547–0.9201])

Decision models for the identification of predictors for adverse outcomes based on biomarkers can increase the quality of care and fast the search for a more tailored therapy. Thus, we aimed at estimating an operable decision model to identify predictors of adverse outcomes through a classification tree analysis. In addition to routinely accessible patient information such as demographics, comorbidities, TfSO and NLR at hospital admission, we included in the model 53 putative biomarkers listed in Additional file 1: Table S1, selected based on whether they had been previously reported as being associated with COVID-19 severity or on their biological action. In addition, we considered in the model for predicting the death also the following clinical variables: age, degree of hypoxemia, concentration of CRP, LDH, and creatinine. These variables were not included in the model predicting the transfer to ICU, since they were the major determinants of clinical decision-making for ICU admission,

The concentration of CXCL10 emerged as the main predictor of both transfer to ICU and death, with overall higher values identifying patients with the higher probability of unfavourable outcome (Figs. 2 and 3). A value of 4782 pg/mL was the optimal cut-point for patient stratification based on the risk of transfer to ICU, and a 16,633 pg/mL threshold maximized separation into patients with low and high risk of death.

**Fig. 4:**
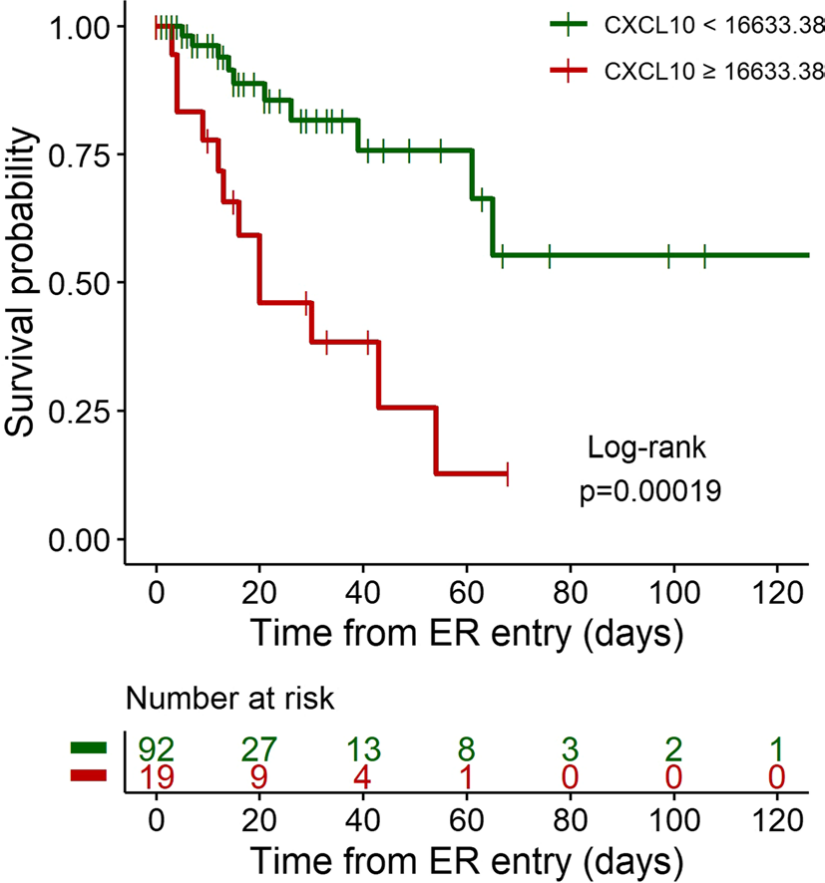
Kaplan–Meier curves of overall survival and logrank test for comparing the two groups obtained from the decision tree analysis predicting the death for CXCL10 levels higher or lower than 16,633 pg/mL

**Fig. 5:**
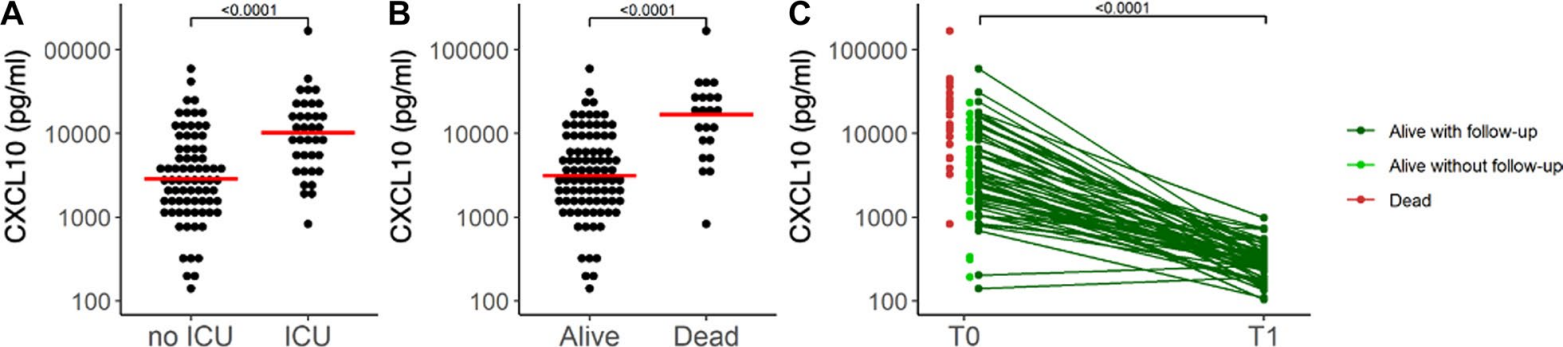
CXCL10 levels (pg/mL) in logarithmic scale of patients: **A** without and with ICU care (n = 75 and 36, respectively), **B** alive and dead (n = 89 and 22, respectively), and **C** alive with a follow-up (n = 57)

Regarding transfer to ICU, NLR at admission and TfSO followed in the hierarchy of prognostic factors identified by the classification tree model (Fig. 2). In contrast, CXCL10 concentration was the only relevant predictor of death (Fig. 3). Survival analysis confirmed that patients with levels of CXCL10 above the identified threshold of 16,633 pg/mL had a significantly higher risk of mortality (p = 0.0002, Fig. 4). Figure 5 depicts the plasma concentration of CXCL10 in COVID-19 survivors and non-survivors and in patients transferred or not to the ICU. CXCL10 concentration was indeed significantly higher in patients with a poor outcome and abated in survivors at follow-up sampling (median [IQR] 23 (20, 40)) after discharge from the hospital (Fig. 5).

**Fig. 6:**
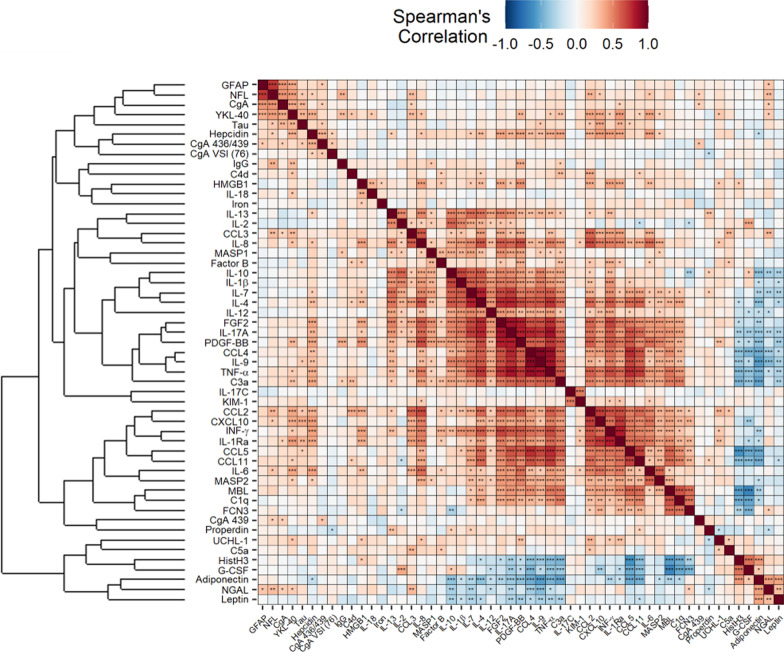
Spearman’s correlations between biomarker levels and hierarchical cluster analysis. The magnitude of each correlation is denoted with a color, whereby the red color indicates a positive correlation and blue color represents a negative correlation, such that the deeper the color, the stronger is the correlation. IgG = anti-SARS-CoV2 spike 1 IgGs. Levels of statistical significance with false discovery rate (FDR) correction are denoted as: p < 0.05, *p < 0.01, ***p < 0.001

We performed a cluster analysis to investigate whether a specific molecular signature involving CXCL10 exists and plays a role in COVID-19. As shown in the heatmap in Fig. 6, CXCL10 clustered with other inflammatory cytokines, such as CCL2, IFN-ϒ, IL-1Ra, CCL5, CCL11, IL-6, MAPS2, MBL and C1q. None of these signals was identified as a predictor of the COVID-19 outcome in the classification tree analysis.

In addition, we correlated the fifty-three putative biomarkers and classical markers in COVID-19 survivors not transferred to the ICU (Additional file 1: Fig. S1) and patients with adverse outcome (ICU or dead) (Additional file 1: Fig. S2). Of note, the levels of CXCL10 were significantly correlated with classical markers only in COVID-19 survivors not transferred to the ICU (Additional file 1: Fig S1). In contrast, this finding was not confirmed in the group of patients with a poor outcome (Additional file 1: Table S2).

## Discussion

Demographic characteristics, comorbidities, clinical manifestations such as hypoxia and laboratory abnormalities including changes in blood cell counts, increased levels of acute phase proteins (i.e. CRP) and cell damage markers (*i.e.* LDH) are associated with severity and outcome in COVID-19. All these features are well represented in our patient cohort (Table 1). In addition, several putative biomarkers have been identified as suitable to profile patients based on the risk of poor outcomes. These include signals evaluated also in our patients, such as inflammatory cytokines (Laing et al. 2020; Rydyznski Moderbacher 2020; Chi et al. 2020; Hue et al. 2020; Chen et al. 2020; Bulow Anderberg 2021; Yang 2020; Mann et al. 2020), complement (Risitano et al. 2020), hepcidin (Nai et al. 2021), neurofilament light chain (Sutter 2020). However, the relative impact of each signal on disease outcome is difficult to pinpoint. To face the challenge of the COVID-19 pandemic we have accumulated an impressive amount of knowledge in a limited time, relying on data collected in emergency conditions, requiring substantially more caution in the analysis than those obtained in high-quality observational studies. Moreover, patients greatly vary in terms of age, comorbidities, base-line treatments, metabolic status etc. All these features impact on the individual inflammatory and immune response and represent a dramatic bias, that must be taken into due account to extract the results that can allow to identify priorities in clinical decision. Consequently, standard statistical approaches are often not sufficient to control for the highly correlated structure between covariates and to account for the many potential confounding factors (Esposito 2020; Cippa, et al. 2021).

These limitations are evident when evaluating the state of the art on potential biomarkers. An ever-increasing number of signals expressed in patients with severe COVID-19 are being identified, but it is extremely difficult to determine which of them can represent a valid addition for the physician and provide information on pathogenetically relevant events in the early stages of the response to SARS-CoV- 2.

We relied on a data-driven approach to tackle these challenges. Specifically, we relied on decision tree models that, within a machine learning perspective, allow for partitioning data into homogeneous subsets determined by hierarchical splits in the covariates (Westreich et al. 2010). Relatively simple decision trees emerged, with a single inflammatory signal, CXCL10, representing the main independent predictor of both adverse outcomes. The association of CXCL10 with clinical severity and outcome is consistent with results of recent studies (Laing et al. 2020; Rydyznski Moderbacher 2020; Chi et al. 2020; Hue et al. 2020; Chen et al. 2020; Bulow Anderberg 2021; Yang 2020; Mann et al. 2020; Huntington et al. 2021). In those studies, a combination of CXCL10 and various other cytokines/chemokines was associated with clinical progression, in line with our observation that CXCL10 is part of an inflammatory signature that comprises several other cytokines and chemokines (Fig. 6). The tree models however consistently reveal that CXCL10 levels per se are sufficient to robustly predict adverse outcomes. The model predicting ICU transfer had an accuracy of 83.7% in our cohort, suggesting that it may be useful to identify patients at increased risk of developing critical illness necessitating ICU admission. These patients would need to be monitored more frequently and intensively and treated promptly and aggressively if clinical conditions change. Of course, the model cannot replace conventional biomarkers for clinical decision making for ICU admission, while being possibly useful in conjunction with these markers.

The robustness of CXCL10 as a biomarker may outweigh its role as a surrogate marker of the COVID-19 cytokine storm width and amplitude. Recent studies have highlighted that the concentration of CXCL10 negatively correlates with the width of the CD4 + and CD8 + T cell repertoire in patients with acute COVID-19 (Rydyznski Moderbacher 2020). It’s worth noting that the authors found out no correlation between levels of CXCL10 and titres of anti-SARS-COV-2 antibodies, confirming our findings (Fig. 6). Balanced activation of acquired SARS-CoV-2 specific immune responses, which include CD4^+^ and CD8^+^ T cells with a memory phenotype and neutralizing antibody responses, is required for host protection in acute COVID-19 (Rydyznski Moderbacher 2020; Mudd and Remy 2021; Sette and Crotty 2021). Notably, CXCL10 plays a non-redundant role in the redistribution of pre-immune memory T cells, i.e. T lymphocytes that circulate with a memory phenotype despite lack of engagement with cognate antigens (Alanio et al. 2018). Our study has limitations. During the first wave of the pandemic blood sampling for research purposes were delayed due to limited resources available for non-clinical activities. For the same reasons, some patients were started on therapy soon after arrival at the ED. Moreover, a relatively small number of patients (n = 111) could be analysed and the findings were obtained in one cohort only, and not internally or externally validated. Further ad hoc multicentre studies involving a substantially greater number of patients are necessary to validate our results and to verify whether the robustness of CXL10 as a marker of COVID-19 outcomes might reflect a direct role of the chemokine in disrupting T cell homeostasis and justify the analysis of this biomarker in conjunction with standard clinical assays.

## Conclusion

Our analysis has been based on data mining and machine-learning techniques such as CART, Random Forest and cluster analysis. The CART in combination with a Random Forest variable pre-selection represents a promising alternative to conventional logistic multiple regression, whose direct application is often precluded in a high-dimensional setting and which shows strong limitations whenever incomplete data are collected. This approach allows a first rigorous assessment of biomarkers of severe COVID-19 outcomes even from databases collected with no design, as it happens in emergency situations, and in presence of several confounding effects. Further ad hoc studies are necessary to verify whether the robustness of CXL10 as a marker of COVID-19 outcomes might reflect a direct role of the chemokine in disrupting T cell homeostasis. CXCL10 value as a biomarker might reflect its action of T cell homeostasis. In this study, we focused on soluble molecules that could be assessed with relative ease in patient plasma, as this could be more realistically transferred into the clinical setting. Given the growing awareness of the role of circulating memory T and B cells in clinical outcomes (Mudd and Remy 2021; Sette and Crotty 2021), information on the acquired immune response would be a valuable addition in further prospective studies.

## Supplementary Information

**Additional file 1.** Additional tables and figures.

## Data Availability

Not applicable.

## References

[CR1] Alanio C (2018). CXCR3/cxcl10 axis shapes tissue distribution of memory phenotype CD8(+) T cells in nonimmunized mice. J Immunol.

[CR2] Arunachalam PS (2020). Systems biological assessment of immunity to mild versus severe COVID-19 infection in humans. Science.

[CR3] Bulow Anderberg S (2021). Increased levels of plasma cytokines and correlations to organ failure and 30-day mortality in critically ill Covid-19 patients. Cytokine.

[CR4] Chen Y (2020). IP-10 and MCP-1 as biomarkers associated with disease severity of COVID-19. Mol Med.

[CR5] Chi Y (2020). Serum cytokine and chemokine profile in relation to the severity of coronavirus disease 2019 in China. J Infect Dis.

[CR6] Ciceri F (2020). Early predictors of clinical outcomes of COVID-19 outbreak in Milan Italy. Clin Immunol.

[CR7] Cippa PE (2021). A data-driven approach to identify risk profiles and protective drugs in COVID-19. Proc Natl Acad Sci U S A.

[CR8] Dan JM (2021). Immunological memory to SARS-CoV-2 assessed for up to 8 months after infection. Science.

[CR9] Danwang C (2020). A meta-analysis of potential biomarkers associated with severity of coronavirus disease 2019 (COVID-19). Biomark Res.

[CR10] Darnell ME, Taylor DR (2006). Evaluation of inactivation methods for severe acute respiratory syndrome coronavirus in noncellular blood products. Transfusion.

[CR11] De Lorenzo R (2020). Residual clinical damage after COVID-19: a retrospective and prospective observational cohort study. PLoS ONE.

[CR12] Del Valle DM (2020). An inflammatory cytokine signature predicts COVID-19 severity and survival. Nat Med.

[CR13] Detsky AS, Naglie G, Krahn MD, Redelmeier DA, Naimark D (1997). Primer on medical decision analysis: part 2–building a tree. Med Decis Making.

[CR14] Esposito A (2020). Chest CT-derived pulmonary artery enlargement at the admission predicts overall survival in COVID-19 patients: insight from 1461 consecutive patients in Italy. Eur Radiol.

[CR15] Farina N (2020). COVID-19: Pharmacology and kinetics of viral clearance. Pharmacol Res.

[CR16] Guneyli S, Atceken Z, Dogan H, Altinmakas E, Atasoy KC (2020). Radiological approach to COVID-19 pneumonia with an emphasis on chest CT. Diagn Interv Radiol.

[CR17] Hue S (2020). Uncontrolled innate and impaired adaptive immune responses in patients with COVID-19 acute respiratory distress syndrome. Am J Respir Crit Care Med.

[CR18] Huntington KE (2021). Cytokine ranking via mutual information algorithm correlates cytokine profiles with presenting disease severity in patients infected with SARS-CoV-2. Elife.

[CR19] Ji P (2020). Association of elevated inflammatory markers and severe COVID-19: a meta-analysis. Medicine (baltimore).

[CR20] Kuri-Cervantes L (2020). Comprehensive mapping of immune perturbations associated with severe COVID-19. Sci Immunol.

[CR21] Kursa MB (2010). Feature selection with the Boruta package. J Stat Softw.

[CR22] Laing AG (2020). A dynamic COVID-19 immune signature includes associations with poor prognosis. Nat Med.

[CR23] Li S (2020). Clinical and pathological investigation of patients with severe COVID-19. JCI Insight.

[CR24] Lucas C (2020). Longitudinal analyses reveal immunological misfiring in severe COVID-19. Nature.

[CR25] Mann ER (2020). Longitudinal immune profiling reveals key myeloid signatures associated with COVID-19. Sci Immunol.

[CR26] Mudd PA, Remy KE (2021). Prolonged adaptive immune activation in COVID-19: implications for maintenance of long-term immunity?. J Clin Invest.

[CR27] Nai A (2021). Hepcidin levels predict Covid-19 severity and mortality in a cohort of hospitalized Italian patients. Am J Hematol.

[CR28] Pauker SG, Kassirer JP (1987). Decision analysis. N Engl J Med.

[CR29] Risitano AM (2020). Complement as a target in COVID-19?. Nat Rev Immunol.

[CR30] Rovere Querini P (2020). Post-COVID-19 follow-up clinic: depicting chronicity of a new disease. Acta Biomed.

[CR31] Rovere-Querini P (2020). Biobanking for COVID-19 research. Panminerva Med.

[CR32] Rydyznski Moderbacher C (2020). Antigen-specific adaptive immunity to SARS-CoV-2 in acute COVID-19 and associations with age and disease severity. Cell.

[CR33] Sette A, Crotty S (2021). Adaptive immunity to SARS-CoV-2 and COVID-19. Cell.

[CR34] Siciliano R (1998). Exploratory versus decision trees. COMPSTAT.

[CR35] Siciliano R, Aria M, D’Ambrosio A, Brito P (2008). Posterior prediction modelling of optimal trees. Proceedings in Computational Statistics (COMPSTAT 2008).

[CR36] Simadibrata DM, Calvin J, Wijaya AD, Ibrahim NAA (2021). Neutrophil-to-lymphocyte ratio on admission to predict the severity and mortality of COVID-19 patients: a meta-analysis. Am J Emerg Med.

[CR37] Sutter R (2020). Serum neurofilament light chain levels in the intensive care unit: comparison between severely ill patients with and without coronavirus disease 2019. Ann Neurol.

[CR38] Tian W (2020). Predictors of mortality in hospitalized COVID-19 patients: a systematic review and meta-analysis. J Med Virol.

[CR39] Tombetti E (2016). Chromogranin-A production and fragmentation in patients with Takayasu arteritis. Arthritis Res Ther.

[CR40] Westreich D, Lessler J, Funk MJ (2010). Propensity score estimation: neural networks, support vector machines, decision trees (CART), and meta-classifiers as alternatives to logistic regression. J Clin Epidemiol.

[CR41] Yang Y (2020). Plasma IP-10 and MCP-3 levels are highly associated with disease severity and predict the progression of COVID-19. J Allergy Clin Immunol.

[CR42] Zhou F (2020). Clinical course and risk factors for mortality of adult inpatients with COVID-19 in Wuhan, China: a retrospective cohort study. Lancet.

