# Supplementary material for: CXCL10 levels at hospital admission predict COVID-19 outcome: hierarchical assessment of 53 putative inflammatory biomarkers in an observational study
Source: Mol Med. 2021 Oct 18;27:129. doi: 10.1186/s10020-021-00390-4 (PMC8521494; doi:10.1186/s10020-021-00390-4)
Supplement: Supplementary file 1 — Additional file 1. Additional tables and figures. [file 10020_2021_390_MOESM1_ESM.docx]

**Supplementary Files**

**CXCL10 levels at hospital admission predict COVID-19 outcome: hierarchical assessment of 53 putative inflammatory biomarkers in an observational study.**

Nicola I. Lorè^1,3^*, Rebecca De Lorenzo^1,2^*, Paola M. V. Rancoita^4^, Federica Cugnata^4^, Alessandra Agresti^5^, Francesco Benedetti^2,6^, Marco E. Bianchi^2,5^, Chiara Bonini^1,2^, Annalisa Capobianco^1^, Caterina Conte^1,2^, Angelo Corti^2,7^, Roberto Furlan^6^, Paola Mantegani^1,3^, Norma Maugeri^1,2^, Clara Sciorati^1^, Fabio Saliu^1,3^, Laura Silvestri^5^, Cristina Tresoldi^8^, Bio Angels for COVID-BioB Study Group, Fabio Ciceri^2,8^, Patrizia Rovere-Querini^1,2^**, Clelia Di Serio^2,4,9^**, Daniela M. Cirillo^1,3^**, Angelo A. Manfredi^1,2^**#

*^1^Division of Immunology, Transplantation and Infectious Diseases, IRCCS San Raffaele Scientific Institute, Milano 20132, Italy; ^2^Vita-Salute San Raffaele University, Milano, Italy; ^3^Emerging Bacterial Pathogens Unit, IRCCS San Raffaele Scientific Institute; ^4^University Centre for Statistics in the Biomedical Sciences (CUSSB), Vita-Salute San Raffaele University, Milan, Italy; ^5^Division of Genetics and Cell Biology, IRCCS San Raffaele Scientific Institute; ^6^Division of Neuroscience, IRCCS San Raffaele Scientific Institute; ^7^Division of Experimental Oncology, IRCCS San Raffaele Scientific Institute.* *^2,8^Hematology and Bone Marrow Transplant, IRCCS San Raffaele Scientific Institute, Milano, Italy,* ^9^ *Faculty of Biomedical Sciences, Swiss University, Lugano,* *Switzerland.*

**Running title: a CXCL10-based risk model for COVID-19**

**Supplementary Fig 1**


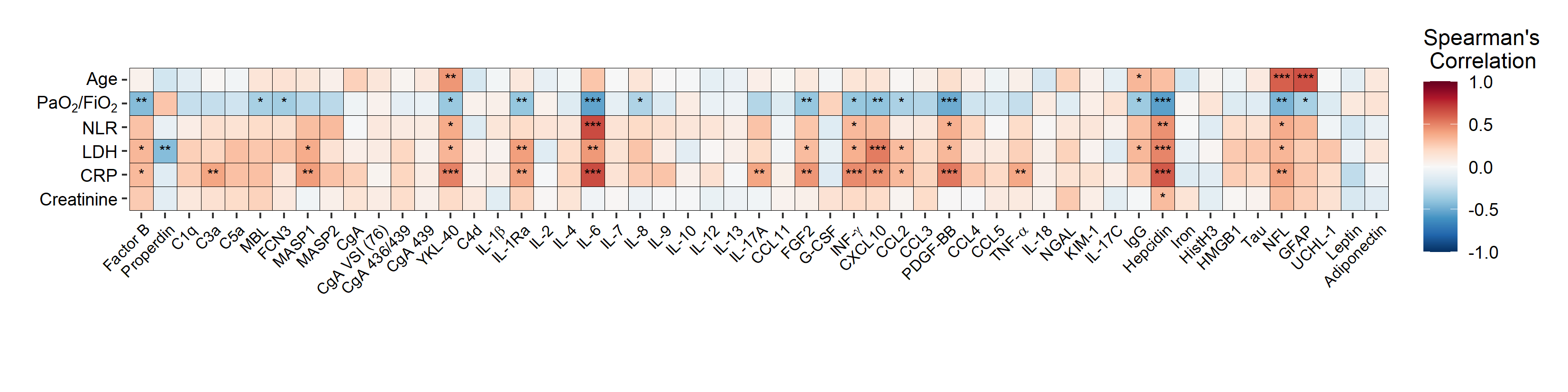


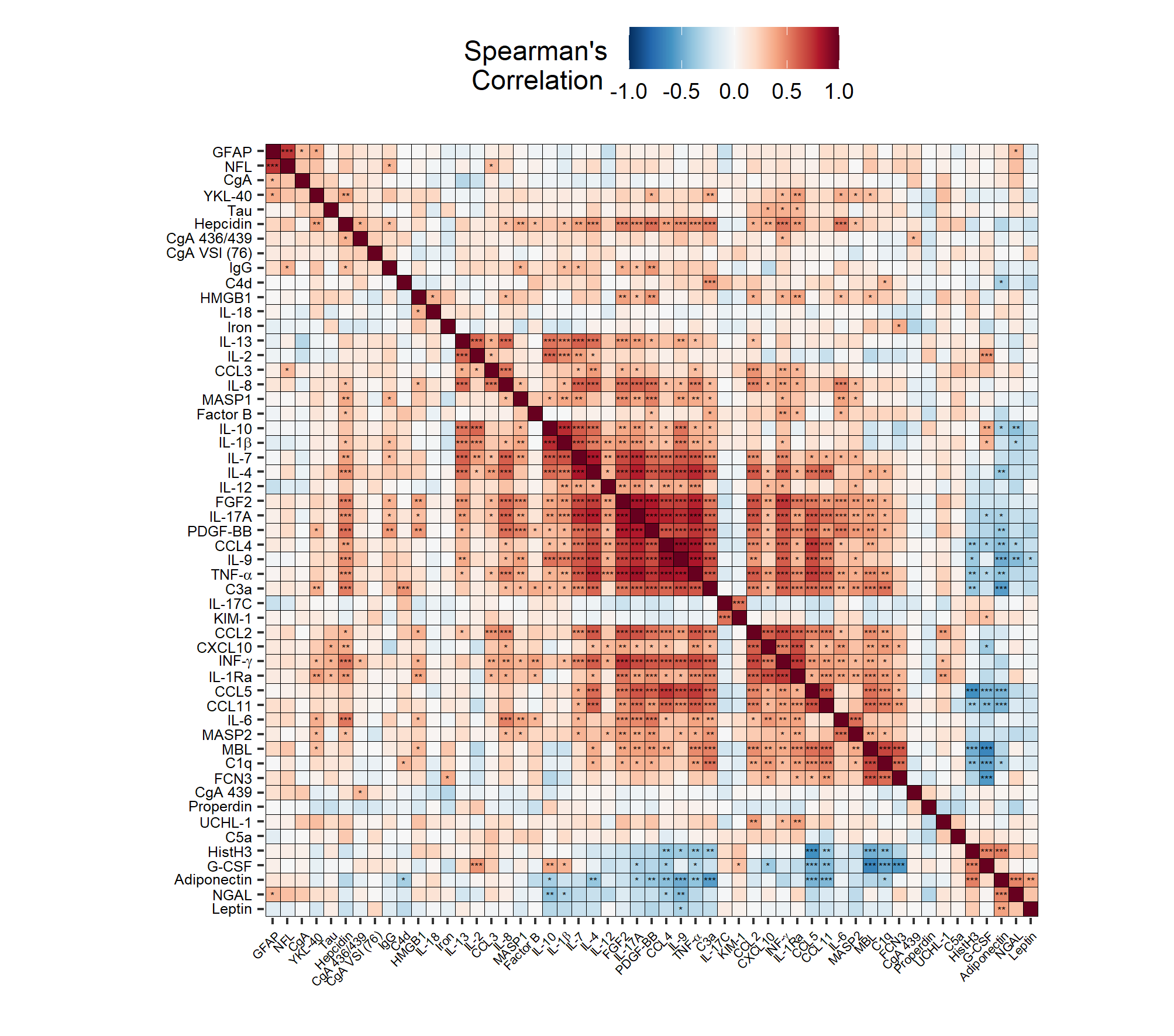


Spearman’s correlations between clinical characteristics and biomarker levels (above) and among biomarker levels (below), within alive patients not transferred to ICU (n=71). The magnitude of each correlation is denoted with a color, whereby the red color indicates a positive correlation and blue color represents a negative correlation, such that the deeper the color, the stronger is the correlation. Levels of statistical significance with false discovery rate (FDR) correction are denoted as: p < 0.05, * p < 0.01, *** p < 0.001. IgG= anti-SARS-CoV2 spike 1 IgGs.

**Supplementary Fig 2**


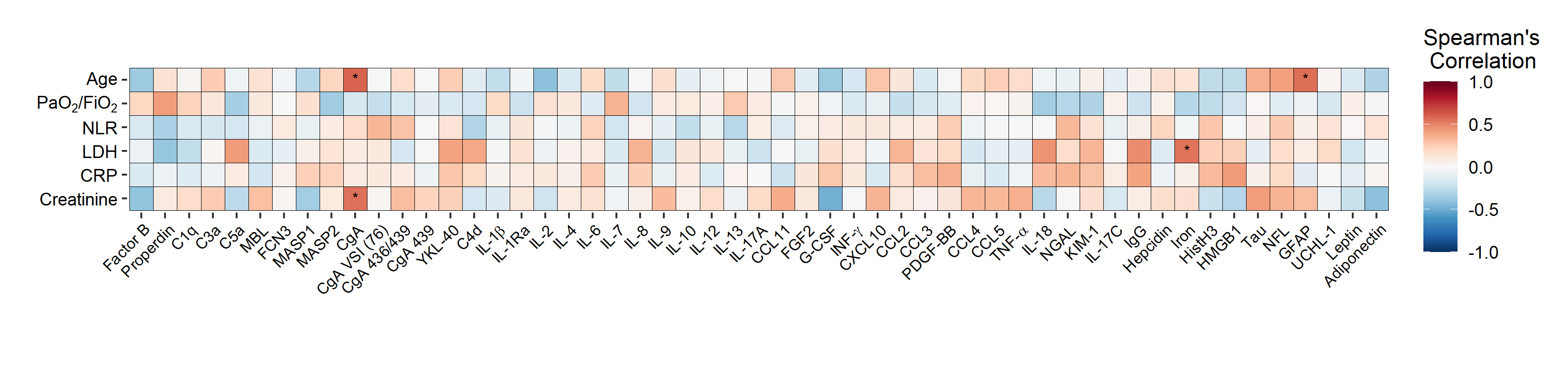


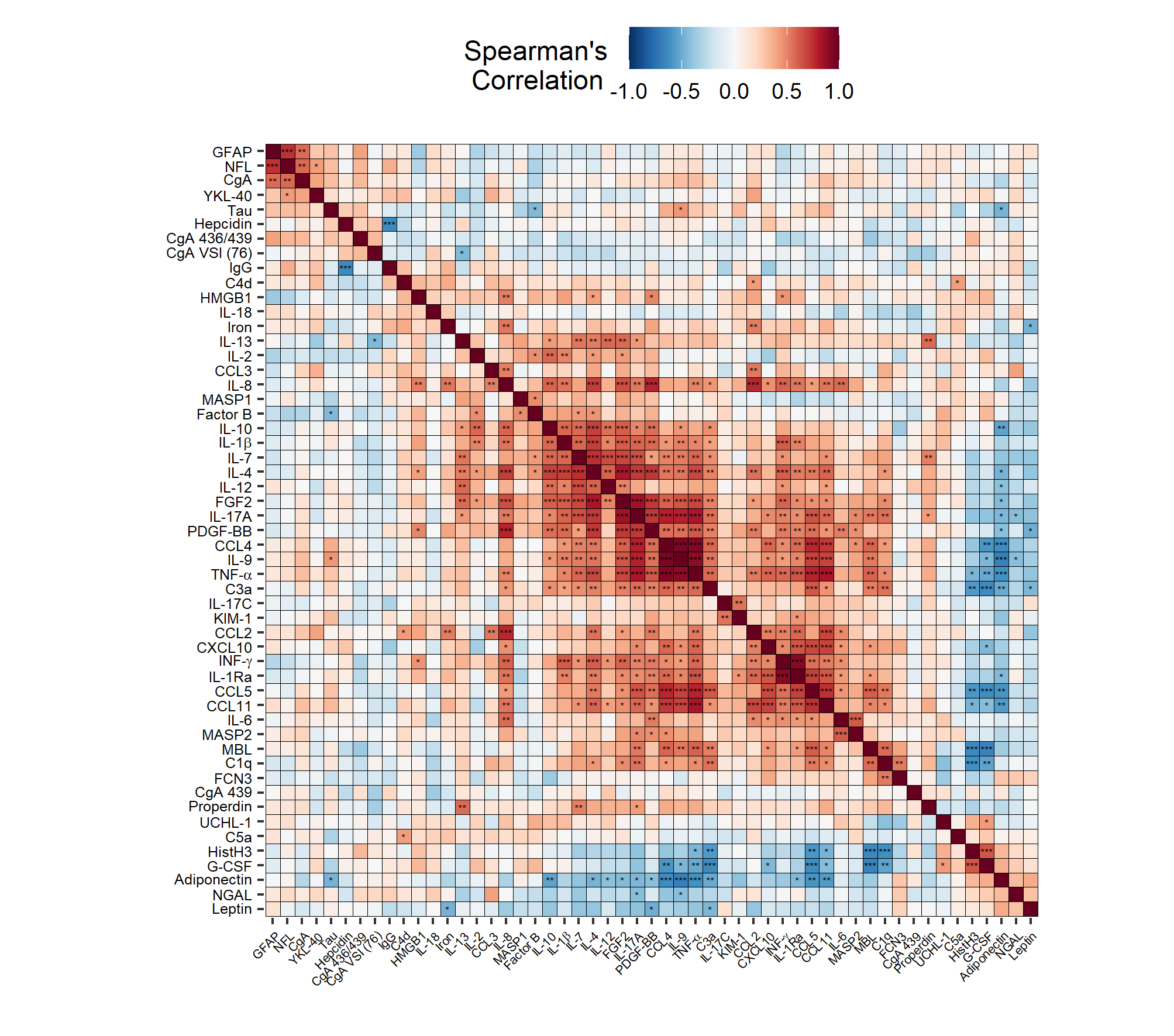


Spearman’s correlations between clinical characteristics and biomarker levels (above) and among biomarker levels (below), within patients dead or transferred to ICU (n=40). The magnitude of each correlation is denoted with a color, whereby the red color indicates a positive correlation and blue color represents a negative correlation, such that the deeper the color, the stronger is the correlation. Levels of statistical significance with false discovery rate (FDR) correction are denoted as: p < 0.05, * p < 0.01, *** p < 0.001. IgG= anti-SARS-CoV2 spike 1 IgGs.

**Table S1**. Biomarkers analyzed in the present study and sources of current knowledge of their role in COVID-19.

| **Biological Functions** | **Biomarkers** | **References related to COVID-19** |
| --- | --- | --- |
| Cytokines, chemokines, adipocytokines and growth factors | IL-1β  IL-1Ra  IL-2  IL-4  IL-6  IL-7  IL-8  IL-9  IL-10  IL-12  IL-13  IL-17  Eotaxin  FGF basic  G-CSF  IFN-γ  CXCL10/IP-10  CCL2/MCP-1  MIP-1α  PDGF-BB  MIP-1β  RANTES  TNF-α  IL-18  NGAL  TIM-1  IL-17C  Leptin  Adiponectin | (1)  (2)  (3)  (4)  (5)  (6)  (7)  (8)  (9)  (9)  (9,10)  (11)  (12)  (13)  (14)  (15,16)  (17,18,19,20)  (21,22,23)  (24)  (25)  (26)  (27)  (28)  (29)  (30)  (31)  Na  (32)  (33) |
| Signals of cell and tissue injury | HMGB1  HistH3 | (34)  Na |
| Markers of the innate/adaptive humoral immune response | Complement Factor B  Properdin  C1q  C3a  C5a  MBL  FCN3  MASP1  MASP2  CgA  CgA VSI  CgA 436/439  CgA 439  YKL-40  C4d | (35)  Na  (36)  (36)  (36)  (37,38)  (37,38)  (37,38)  (37,38)  Na  Na  Na  Na  (39)  (40) |
| Iron metabolism markers | Hepcidin  Iron | (41)  (42) |
| Neuroendocrine molecules | Tau  NFL  GFAP  UCHL-1 | (43,44)  (45,46)  (47)  Na |

REFERENCES

1. Bell LCK, Meydan C, Kim J, Foox J, Butler D, Mason CE, Shapira SD, Noursadeghi M, Pollara G. Transcriptional response modules characterize IL-1β and IL-6 activity in COVID-19. iScience. 2021 Jan 22;24(1):101896. doi: 10.1016/j.isci.2020.101896. Epub 2020 Dec 7. PMID: 33319166; PMCID: PMC7721347
2. Zhao Y, Qin L, Zhang P, Li K, Liang L, Sun J, Xu B, Dai Y, Li X, Zhang C, Peng Y, Feng Y, Li A, Hu Z, Xiang H, Ogg G, Ho LP, McMichael A, Jin R, Knight JC, Dong T, Zhang Y. Longitudinal COVID-19 profiling associates IL-1RA and IL-10 with disease severity and RANTES with mild disease. JCI Insight. 2020 Jul 9;5(13):e139834. doi: 10.1172/jci.insight.139834. PMID: 32501293; PMCID: PMC7406242.
3. Costela-Ruiz VJ, Illescas-Montes R, Puerta-Puerta JM, Ruiz C, Melguizo-Rodríguez L. SARS-CoV-2 infection: The role of cytokines in COVID-19 disease. Cytokine Growth Factor Rev. 2020 Aug;54:62-75. doi: 10.1016/j.cytogfr.2020.06.001. Epub 2020 Jun 2. PMID: 32513566; PMCID: PMC7265853.
4. Vaz de Paula CB, de Azevedo MLV, Nagashima S, Martins APC, Malaquias MAS, Miggiolaro AFRDS, da Silva Motta Júnior J, Avelino G, do Carmo LAP, Carstens LB, de Noronha L. IL-4/IL-13 remodeling pathway of COVID-19 lung injury. Sci Rep. 2020 Oct 29;10(1):18689. doi: 10.1038/s41598-020-75659-5. PMID: 33122784; PMCID: PMC7596721.
5. Bermejo-Martin JF, González-Rivera M, Almansa R, Micheloud D, Tedim AP, Domínguez-Gil M, Resino S, Martín-Fernández M, Ryan Murua P, Pérez-García F, Tamayo L, Lopez-Izquierdo R, Bustamante E, Aldecoa C, Gómez JM, Rico-Feijoo J, Orduña A, Méndez R, Fernández Natal I, Megías G, González-Estecha M, Carriedo D, Doncel C, Jorge N, Ortega A, de la Fuente A, Del Campo F, Fernández-Ratero JA, Trapiello W, González-Jiménez P, Ruiz G, Kelvin AA, Ostadgavahi AT, Oneizat R, Ruiz LM, Miguéns I, Gargallo E, Muñoz I, Pelegrin S, Martín S, García Olivares P, Cedeño JA, Ruiz Albi T, Puertas C, Berezo JÁ, Renedo G, Herrán R, Bustamante-Munguira J, Enríquez P, Cicuendez R, Blanco J, Abadia J, Gómez Barquero J, Mamolar N, Blanca-López N, Valdivia LJ, Fernández Caso B, Mantecón MÁ, Motos A, Fernandez-Barat L, Ferrer R, Barbé F, Torres A, Menéndez R, Eiros JM, Kelvin DJ. Viral RNA load in plasma is associated with critical illness and a dysregulated host response in COVID-19. Crit Care. 2020 Dec 14;24(1):691. doi: 10.1186/s13054-020-03398-0. PMID: 33317616; PMCID: PMC7734467.
6. Huang C, Wang Y, Li X, Ren L, Zhao J, Hu Y, Zhang L, Fan G, Xu J, Gu X, Cheng Z, Yu T, Xia J, Wei Y, Wu W, Xie X, Yin W, Li H, Liu M, Xiao Y, Gao H, Guo L, Xie J, Wang G, Jiang R, Gao Z, Jin Q, Wang J, Cao B. Clinical features of patients infected with 2019 novel coronavirus in Wuhan, China. Lancet. 2020 Feb 15;395(10223):497-506. doi: 10.1016/S0140-6736(20)30183-5. Epub 2020 Jan 24. Erratum in: Lancet. 2020 Jan 30;: PMID: 31986264; PMCID: PMC7159299.
7. Liu QQ, Cheng A, Wang Y, Li H, Hu L, Zhao X, Wang T, He F. Cytokines and their relationship with the severity and prognosis of coronavirus disease 2019 (COVID-19): a retrospective cohort study. BMJ Open. 2020 Nov 30;10(11):e041471. doi: 10.1136/bmjopen-2020-041471. PMID: 33257492; PMCID: PMC7705426.
8. Ghazavi A, Ganji A, Keshavarzian N, Rabiemajd S, Mosayebi G. Cytokine profile and disease severity in patients with COVID-19. Cytokine. 2021 Jan;137:155323. doi: 10.1016/j.cyto.2020.155323. Epub 2020 Sep 30. PMID: 33045526; PMCID: PMC7524708.
9. Costela-Ruiz VJ, Illescas-Montes R, Puerta-Puerta JM, Ruiz C, Melguizo-Rodríguez L. SARS-CoV-2 infection: The role of cytokines in COVID-19 disease. Cytokine Growth Factor Rev. 2020 Aug;54:62-75. doi: 10.1016/j.cytogfr.2020.06.001. Epub 2020 Jun 2. PMID: 32513566; PMCID: PMC7265853.
10. Vaz de Paula CB, de Azevedo MLV, Nagashima S, Martins APC, Malaquias MAS, Miggiolaro AFRDS, da Silva Motta Júnior J, Avelino G, do Carmo LAP, Carstens LB, de Noronha L. IL-4/IL-13 remodeling pathway of COVID-19 lung injury. Sci Rep. 2020 Oct 29;10(1):18689. doi: 10.1038/s41598-020-75659-5. PMID: 33122784; PMCID: PMC7596721.
11. Ghazavi A, Ganji A, Keshavarzian N, Rabiemajd S, Mosayebi G. Cytokine profile and disease severity in patients with COVID-19. Cytokine. 2021 Jan;137:155323. doi: 10.1016/j.cyto.2020.155323. Epub 2020 Sep 30. PMID: 33045526; PMCID: PMC7524708.
12. Horspool AM, Kieffer T, Russ BP, DeJong MA, Wolf MA, Karakiozis JM, Hickey BJ, Fagone P, Tacker DH, Bevere JR, Martinez I, Barbier M, Perrotta PL, Damron FH. Interplay of Antibody and Cytokine Production Reveals CXCL13 as a Potential Novel Biomarker of Lethal SARS-CoV-2 Infection. mSphere. 2021 Jan 20;6(1):e01324-20. doi: 10.1128/mSphere.01324-20. PMID: 33472985.
13. Meini S, Giani T, Tascini C. Intussusceptive angiogenesis in Covid-19: hypothesis on the significance and focus on the possible role of FGF2. Mol Biol Rep. 2020 Oct;47(10):8301-8304. doi: 10.1007/s11033-020-05831-7. Epub 2020 Sep 12. PMID: 32920756; PMCID: PMC7486971.
14. Jøntvedt Jørgensen M, Holter JC, Christensen EE, Schjalm C, Tonby K, Pischke SE, Jenum S, Skeie LG, Nur S, Lind A, Opsand H, Enersen TB, Grøndahl R, Hermann A, Dudman S, Muller F, Ueland T, Mollnes TE, Aukrust P, Heggelund L, Holten AR, Dyrhol-Riise AM. Increased interleukin-6 and macrophage chemoattractant protein-1 are associated with respiratory failure in COVID-19. Sci Rep. 2020 Dec 10;10(1):21697. doi: 10.1038/s41598-020-78710-7. PMID: 33303843; PMCID: PMC7729930.
15. Costela-Ruiz VJ, Illescas-Montes R, Puerta-Puerta JM, Ruiz C, Melguizo-Rodríguez L. SARS-CoV-2 infection: The role of cytokines in COVID-19 disease. Cytokine Growth Factor Rev. 2020 Aug;54:62-75. doi: 10.1016/j.cytogfr.2020.06.001. Epub 2020 Jun 2. PMID: 32513566; PMCID: PMC7265853.
16. Ghazavi A, Ganji A, Keshavarzian N, Rabiemajd S, Mosayebi G. Cytokine profile and disease severity in patients with COVID-19. Cytokine. 2021 Jan;137:155323. doi: 10.1016/j.cyto.2020.155323. Epub 2020 Sep 30. PMID: 33045526; PMCID: PMC7524708.
17. Blot M, Jacquier M, Aho Glele LS, Beltramo G, Nguyen M, Bonniaud P, Prin S, Andreu P, Bouhemad B, Bour JB, Binquet C, Piroth L, Pais de Barros JP, Masson D, Quenot JP, Charles PE; Pneumochondrie study group. CXCL10 could drive longer duration of mechanical ventilation during COVID-19 ARDS. Crit Care. 2020 Nov 2;24(1):632. doi: 10.1186/s13054-020-03328-0. PMID: 33138839; PMCID: PMC7604548.
18. Chen Y, Wang J, Liu C, Su L, Zhang D, Fan J, Yang Y, Xiao M, Xie J, Xu Y, Li Y, Zhang S. IP-10 and MCP-1 as biomarkers associated with disease severity of COVID-19. Mol Med. 2020 Oct 29;26(1):97. doi: 10.1186/s10020-020-00230-x. PMID: 33121429; PMCID: PMC7594996.
19. Hue S, Beldi-Ferchiou A, Bendib I, Surenaud M, Fourati S, Frapard T, Rivoal S, Razazi K, Carteaux G, Delfau-Larue MH, Mekontso-Dessap A, Audureau E, de Prost N. Uncontrolled Innate and Impaired Adaptive Immune Responses in Patients with COVID-19 Acute Respiratory Distress Syndrome. Am J Respir Crit Care Med. 2020 Dec 1;202(11):1509-1519. doi: 10.1164/rccm.202005-1885OC. PMID: 32866033; PMCID: PMC7706149.
20. Rydyznski Moderbacher C, Ramirez SI, Dan JM, Grifoni A, Hastie KM, Weiskopf D, Belanger S, Abbott RK, Kim C, Choi J, Kato Y, Crotty EG, Kim C, Rawlings SA, Mateus J, Tse LPV, Frazier A, Baric R, Peters B, Greenbaum J, Ollmann Saphire E, Smith DM, Sette A, Crotty S. Antigen-Specific Adaptive Immunity to SARS-CoV-2 in Acute COVID-19 and Associations with Age and Disease Severity. Cell. 2020 Nov 12;183(4):996-1012.e19. doi: 10.1016/j.cell.2020.09.038. Epub 2020 Sep 16. PMID: 33010815; PMCID: PMC7494270.
21. Costela-Ruiz VJ, Illescas-Montes R, Puerta-Puerta JM, Ruiz C, Melguizo-Rodríguez L. SARS-CoV-2 infection: The role of cytokines in COVID-19 disease. Cytokine Growth Factor Rev. 2020 Aug;54:62-75. doi: 10.1016/j.cytogfr.2020.06.001. Epub 2020 Jun 2. PMID: 32513566; PMCID: PMC7265853.
22. Abers MS, Delmonte OM, Ricotta EE, Fintzi J, Fink DL, de Jesus AAA, Zarember KA, Alehashemi S, Oikonomou V, Desai JV, Canna SW, Shakoory B, Dobbs K, Imberti L, Sottini A, Quiros-Roldan E, Castelli F, Rossi C, Brugnoni D, Biondi A, Bettini LR, D'Angio' M, Bonfanti P, Castagnoli R, Montagna D, Licari A, Marseglia GL, Gliniewicz EF, Shaw E, Kahle DE, Rastegar AT, Stack M, Myint-Hpu K, Levinson SL, DiNubile MJ, Chertow DW, Burbelo PD, Cohen JI, Calvo KR, Tsang JS; NIAID COVID-19 Consortium, Su HC, Gallin JI, Kuhns DB, Goldbach-Mansky R, Lionakis MS, Notarangelo LD. An immune-based biomarker signature is associated with mortality in COVID-19 patients. JCI Insight. 2021 Jan 11;6(1):144455. doi: 10.1172/jci.insight.144455. PMID: 33232303.
23. Hue S, Beldi-Ferchiou A, Bendib I, Surenaud M, Fourati S, Frapard T, Rivoal S, Razazi K, Carteaux G, Delfau-Larue MH, Mekontso-Dessap A, Audureau E, de Prost N. Uncontrolled Innate and Impaired Adaptive Immune Responses in Patients with COVID-19 Acute Respiratory Distress Syndrome. Am J Respir Crit Care Med. 2020 Dec 1;202(11):1509-1519. doi: 10.1164/rccm.202005-1885OC. PMID: 32866033; PMCID: PMC7706149.
24. Huang C, Wang Y, Li X, Ren L, Zhao J, Hu Y, Zhang L, Fan G, Xu J, Gu X, Cheng Z, Yu T, Xia J, Wei Y, Wu W, Xie X, Yin W, Li H, Liu M, Xiao Y, Gao H, Guo L, Xie J, Wang G, Jiang R, Gao Z, Jin Q, Wang J, Cao B. Clinical features of patients infected with 2019 novel coronavirus in Wuhan, China. Lancet. 2020 Feb 15;395(10223):497-506. doi: 10.1016/S0140-6736(20)30183-5. Epub 2020 Jan 24. Erratum in: Lancet. 2020 Jan 30;: PMID: 31986264; PMCID: PMC7159299.
25. Young BE, Ong SWX, Ng LFP, Anderson DE, Chia WN, Chia PY, Ang LW, Mak TM, Kalimuddin S, Chai LYA, Pada S, Tan SY, Sun L, Parthasarathy P, Fong SW, Chan YH, Tan CW, Lee B, Rötzschke O, Ding Y, Tambyah P, Low JGH, Cui L, Barkham T, Lin RTP, Leo YS, Renia L, Wang LF, Lye DC; Singapore 2019 Novel Coronavirus Outbreak Research team. Viral dynamics and immune correlates of COVID-19 disease severity. Clin Infect Dis. 2020 Aug 28:ciaa1280. doi: 10.1093/cid/ciaa1280. Epub ahead of print. PMID: 32856707; PMCID: PMC7499509.
26. Xiong Y, Liu Y, Cao L, Wang D, Guo M, Jiang A, Guo D, Hu W, Yang J, Tang Z, Wu H, Lin Y, Zhang M, Zhang Q, Shi M, Liu Y, Zhou Y, Lan K, Chen Y. Transcriptomic characteristics of bronchoalveolar lavage fluid and peripheral blood mononuclear cells in COVID-19 patients. Emerg Microbes Infect. 2020 Dec;9(1):761-770. doi: 10.1080/22221751.2020.1747363. PMID: 32228226; PMCID: PMC7170362.
27. Hue S, Beldi-Ferchiou A, Bendib I, Surenaud M, Fourati S, Frapard T, Rivoal S, Razazi K, Carteaux G, Delfau-Larue MH, Mekontso-Dessap A, Audureau E, de Prost N. Uncontrolled Innate and Impaired Adaptive Immune Responses in Patients with COVID-19 Acute Respiratory Distress Syndrome. Am J Respir Crit Care Med. 2020 Dec 1;202(11):1509-1519. doi: 10.1164/rccm.202005-1885OC. PMID: 32866033; PMCID: PMC7706149.
28. Huang C, Wang Y, Li X, Ren L, Zhao J, Hu Y, Zhang L, Fan G, Xu J, Gu X, Cheng Z, Yu T, Xia J, Wei Y, Wu W, Xie X, Yin W, Li H, Liu M, Xiao Y, Gao H, Guo L, Xie J, Wang G, Jiang R, Gao Z, Jin Q, Wang J, Cao B. Clinical features of patients infected with 2019 novel coronavirus in Wuhan, China. Lancet. 2020 Feb 15;395(10223):497-506. doi: 10.1016/S0140-6736(20)30183-5. Epub 2020 Jan 24. Erratum in: Lancet. 2020 Jan 30;: PMID: 31986264; PMCID: PMC7159299.
29. Rodrigues TS, de Sá KSG, Ishimoto AY, Becerra A, Oliveira S, Almeida L, Gonçalves AV, Perucello DB, Andrade WA, Castro R, Veras FP, Toller-Kawahisa JE, Nascimento DC, de Lima MHF, Silva CMS, Caetite DB, Martins RB, Castro IA, Pontelli MC, de Barros FC, do Amaral NB, Giannini MC, Bonjorno LP, Lopes MIF, Santana RC, Vilar FC, Auxiliadora-Martins M, Luppino-Assad R, de Almeida SCL, de Oliveira FR, Batah SS, Siyuan L, Benatti MN, Cunha TM, Alves-Filho JC, Cunha FQ, Cunha LD, Frantz FG, Kohlsdorf T, Fabro AT, Arruda E, de Oliveira RDR, Louzada-Junior P, Zamboni DS. Inflammasomes are activated in response to SARS-CoV-2 infection and are associated with COVID-19 severity in patients. J Exp Med. 2021 Mar 1;218(3):e20201707. doi: 10.1084/jem.20201707. PMID: 33231615; PMCID: PMC7684031.
30. Abers MS, Delmonte OM, Ricotta EE, Fintzi J, Fink DL, de Jesus AAA, Zarember KA, Alehashemi S, Oikonomou V, Desai JV, Canna SW, Shakoory B, Dobbs K, Imberti L, Sottini A, Quiros-Roldan E, Castelli F, Rossi C, Brugnoni D, Biondi A, Bettini LR, D'Angio' M, Bonfanti P, Castagnoli R, Montagna D, Licari A, Marseglia GL, Gliniewicz EF, Shaw E, Kahle DE, Rastegar AT, Stack M, Myint-Hpu K, Levinson SL, DiNubile MJ, Chertow DW, Burbelo PD, Cohen JI, Calvo KR, Tsang JS; NIAID COVID-19 Consortium, Su HC, Gallin JI, Kuhns DB, Goldbach-Mansky R, Lionakis MS, Notarangelo LD. An immune-based biomarker signature is associated with mortality in COVID-19 patients. JCI Insight. 2021 Jan 11;6(1):144455. doi: 10.1172/jci.insight.144455. PMID: 33232303.
31. Luther T, Bülow-Anderberg S, Larsson A, Rubertsson S, Lipcsey M, Frithiof R, Hultström M. COVID-19 patients in intensive care develop predominantly oliguric acute kidney injury. Acta Anaesthesiol Scand. 2020 Nov 15:10.1111/aas.13746. doi: 10.1111/aas.13746. Epub ahead of print. PMID: 33190222; PMCID: PMC7753792.
32. Wang J, Xu Y, Zhang X, Wang S, Peng Z, Guo J, Jiang H, Liu J, Xie Y, Wang J, Li X, Liao J, Wan C, Yu L, Hu J, Liu B, Liu Z. Leptin correlates with monocytes activation and severe condition in COVID-19 patients. J Leukoc Biol. 2021 Jan 6. doi: 10.1002/JLB.5HI1020-704R. Epub ahead of print. PMID: 33404078.
33. Marasca C, Fabbrocini G, Barrea L, Capasso G, DI Guida A, Cinelli E, Fontanella G. Endocrinological disorders and inflammatory skin diseases during COVID-19 outbreak: a review of the literature. Minerva Endocrinol. 2020 Dec;45(4):345-353. doi: 10.23736/S0391-1977.20.03248-4. Epub 2020 Sep 24. PMID: 32969629.
34. Chen R, Huang Y, Quan J, Liu J, Wang H, Billiar TR, Lotze MT, Zeh HJ, Kang R, Tang D. HMGB1 as a potential biomarker and therapeutic target for severe COVID-19. Heliyon. 2020 Dec 7;6(12):e05672. doi: 10.1016/j.heliyon.2020.e05672. PMID: 33313438; PMCID: PMC7720697.
35. Kurtovic L, Beeson JG. Complement Factors in COVID-19 Therapeutics and Vaccines. Trends Immunol. 2020 Dec 13:S1471-4906(20)30283-0. doi: 10.1016/j.it.2020.12.002. Epub ahead of print. PMID: 33402318; PMCID: PMC7733687.
36. Lo MW, Kemper C, Woodruff TM. COVID-19: Complement, Coagulation, and Collateral Damage. J Immunol. 2020 Sep 15;205(6):1488-1495. doi: 10.4049/jimmunol.2000644. Epub 2020 Jul 22. PMID: 32699160; PMCID: PMC7484432.
37. Chatterjee SK, Saha S, Munoz MNM. Molecular Pathogenesis, Immunopathogenesis and Novel Therapeutic Strategy Against COVID-19. Front Mol Biosci. 2020 Aug 11;7:196. doi: 10.3389/fmolb.2020.00196. PMID: 32850977; PMCID: PMC7431665.
38. Świerzko AS, Cedzyński M. The Influence of the Lectin Pathway of Complement Activation on Infections of the Respiratory System. Front Immunol. 2020 Oct 21;11:585243. doi: 10.3389/fimmu.2020.585243. PMID: 33193407; PMCID: PMC7609860.
39. Zeng HL, Chen D, Yan J, Yang Q, Han QQ, Li SS, Cheng L. Proteomic characteristics of bronchoalveolar lavage fluid in critical COVID-19 patients. FEBS J. 2020 Oct 24. doi: 10.1111/febs.15609. Epub ahead of print. PMID: 33098359.
40. Magro C, Mulvey JJ, Berlin D, Nuovo G, Salvatore S, Harp J, Baxter-Stoltzfus A, Laurence J. Complement associated microvascular injury and thrombosis in the pathogenesis of severe COVID-19 infection: A report of five cases. Transl Res. 2020 Jun;220:1-13. doi: 10.1016/j.trsl.2020.04.007. Epub 2020 Apr 15. PMID: 32299776; PMCID: PMC7158248.
41. Nai A, Lorè NI, Pagani A, De Lorenzo R, Di Modica S, Saliu F, Cirillo DM, Rovere-Querini P, Manfredi AA, Silvestri L. Hepcidin levels predict Covid-19 severity and mortality in a cohort of hospitalized Italian patients. Am J Hematol. 2021 Jan;96(1):E32-E35. doi: 10.1002/ajh.26027. Epub 2020 Nov 3. PMID: 33075189.
42. Hippchen T, Altamura S, Muckenthaler MU, Merle U. Hypoferremia is Associated With Increased Hospitalization and Oxygen Demand in COVID-19 Patients. Hemasphere. 2020 Nov 10;4(6):e492. doi: 10.1097/HS9.0000000000000492. PMID: 33205000.
43. Caporale N, Testa G. COVID-19 lessons from the dish: Dissecting CNS manifestations through brain organoids. EMBO J. 2021 Jan 15;40(2):e107213. doi: 10.15252/embj.2020107213. Epub 2020 Dec 2. PMID: 33175425; PMCID: PMC7809698.
44. Pilotto A, Masciocchi S, Volonghi I, De Giuli V, Caprioli F, Mariotto S, Ferrari S, Bozzetti S, Imarisio A, Risi B, Premi E, Benussi A, Focà E, Castelli F, Zanusso G, Monaco S, Stefanelli P, Gasparotti R, Zekeridou A, McKeon A, Ashton NJ, Blennov K, Zetterberg H, Padovani A. SARS-CoV-2 encephalitis is a cytokine release syndrome: evidences from cerebrospinal fluid analyses. Clin Infect Dis. 2021 Jan 4:ciaa1933. doi: 10.1093/cid/ciaa1933. Epub ahead of print. PMID: 33395482
45. Pilotto A, Masciocchi S, Volonghi I, De Giuli V, Caprioli F, Mariotto S, Ferrari S, Bozzetti S, Imarisio A, Risi B, Premi E, Benussi A, Focà E, Castelli F, Zanusso G, Monaco S, Stefanelli P, Gasparotti R, Zekeridou A, McKeon A, Ashton NJ, Blennov K, Zetterberg H, Padovani A. SARS-CoV-2 encephalitis is a cytokine release syndrome: evidences from cerebrospinal fluid analyses. Clin Infect Dis. 2021 Jan 4:ciaa1933. doi: 10.1093/cid/ciaa1933. Epub ahead of print. PMID: 33395482.
46. Kanberg N, Ashton NJ, Andersson LM, Yilmaz A, Lindh M, Nilsson S, Price RW, Blennow K, Zetterberg H, Gisslén M. Neurochemical evidence of astrocytic and neuronal injury commonly found in COVID-19. Neurology. 2020 Sep 22;95(12):e1754-e1759. doi: 10.1212/WNL.0000000000010111. Epub 2020 Jun 16. PMID: 32546655.
47. Kanberg N, Ashton NJ, Andersson LM, Yilmaz A, Lindh M, Nilsson S, Price RW, Blennow K, Zetterberg H, Gisslén M. Neurochemical evidence of astrocytic and neuronal injury commonly found in COVID-19. Neurology. 2020 Sep 22;95(12):e1754-e1759. doi: 10.1212/WNL.0000000000010111. Epub 2020 Jun 16. PMID: 32546655.
